# Low dimensional nanomaterials for treating acute kidney injury

**DOI:** 10.1186/s12951-022-01712-2

**Published:** 2022-12-01

**Authors:** Yuanpeng Nie, Liying Wang, Xinru You, Xiaohua Wang, Jun Wu, Zhihua Zheng

**Affiliations:** 1 Department of Nephrology, Center of Kidney and Urology, The Seventh Affiliated Hospital, Sun Yat-sen University, Shenzhen, 518107 China; 2 Department of Hematology, The Seventh Affiliated Hospital, Sun Yat-sen University, Shenzhen, 518107 China; 3 Department of Pediatrics, The Seventh Affiliated Hospital, Sun Yat-sen University, Shenzhen, 518107 China; 4 Bioscience and Biomedical Engineering Thrust, The Hong Kong University of Science and Technology (Guangzhou), Nansha, Guangzhou, 511400 China; 5 Division of Life Science, The Hong Kong University of Science and Technology, Hong Kong SAR, China

**Keywords:** Low dimension, Nanomaterials, Treatment, Acute kidney injury

## Abstract

Acute kidney injury (AKI) is one of the most common severe complications among hospitalized patients. In the absence of specific drugs to treat AKI, hemodialysis remains the primary clinical treatment for AKI patients. AKI treatment has received significant attention recently due to the excellent drug delivery capabilities of low-dimensional nanomaterials (LDNs) and their unique therapeutic effects. Diverse LDNs have been proposed to treat AKI, with promising results and the potential for future clinical application. This article aims to provide an overview of the pathogenesis of AKI and the recent advances in the treatment of AKI using different types of LDNs. In addition, it is intended to provide theoretical support for the design of LDNs and implications for AKI treatment.

**Graphical Abstract:**

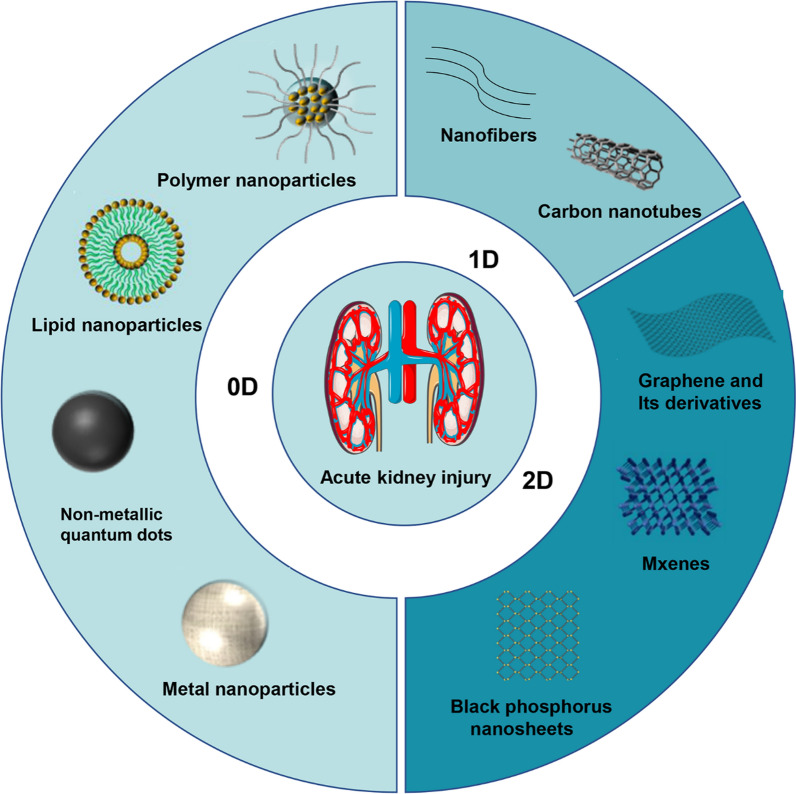

## Introduction

Over the past few decades, acute kidney injury (AKI) incidence has increased due to aging populations, changing dietary habits, and other related factors [1]. According to a global survey, the incidence of AKI in hospitalized adult patients ranged from 3.0 to 18.3% [2]. Worldwide, one in five adults and one in three children experience AKI during hospitalization, and close to 40% of intensive care patients are affected by AKI [1].Severe AKI also induces inflammatory reactions that affect organs throughout the body. Moreover, the renal function of patients has different degrees of damage, and it is difficult to fully recover, which makes these patients have the risk of long-term morbidity and mortality. Some treatments, including dialysis for AKI, are mainly supportive. Early initiation of dialysis may yield benefits by avoiding hypervolemia, hyperkalemia, promoting toxin elimination, establishing acid–base homeostasis, and preventing other complications associated with AKI. Although hemodialysis is commonly used clinically to treat AKI, there are still some problems, and early initiation of dialysis may unnecessarily expose some patients to potential harm because some patients can spontaneously recover renal function.

AKI is generally defined as a sudden decrease in renal function that may lead to azotemia [3]. It is also important to note that this vague definition and the difficulty of accurately diagnosing AKI in different patient populations can significantly impact the prognosis for AKI. There are some standard agreements, and efforts have been made to produce uniform and consistent definitions and standardized diagnostic criteria [3–5]. The latest diagnostic criteria for AKI are based on elevated serum creatinine (sCr) or decreased urine volume. However, sCr is not an ideal biomarker for AKI because it does not rise until 24–72 h after kidney injury and can be influenced by various non-renal factors, such as age, sex, and dietary intake. Additionally, oliguria may be nonspecific in AKI. Therefore, researchers are actively looking for new biological markers for early diagnosis of AKI, including neutrophil gelatinase-associated lipocalin, semaphorin-3A, renal injury inhibition C, kidney injury molecule-1, netrin-1, liver-type fatty-acid binding protein, and metalloproteinase tissue inhibitors-2 [6–10].

The lack of a gold standard for AKI diagnosis means that estimates of AKI incidence may be inaccurate. Renal failure is usually a precursor to multiple organ dysfunction and systemic disease. Diagnosis is often delayed; unfortunately, it is more likely to be challenging to treat AKI [11]. Some treatments, including RRT for AKI, are mainly supportive and have not yet established interventions.

Nanotechnology represents a very promising therapeutic strategy. In recent years, low-dimensional nanomaterials (LDNs) have attracted significant interest from the scientific community [12–14]. LDNs, such as quantum dots, nanowires, and nanosheets, possess general characteristics that are less than three-dimensional nanomaterials, including large surface area, abundant binding sites, and excellent cell permeability, making them ideal materials for drug and biomolecule payloads, specific surface modifications, and targeted cell delivery [15]. Adequately formulated LDNs can treat renal disease more effectively than small molecule medicines.

This article reviews the pathophysiology of AKI, including oxidative stress, inflammation, mitochondrial dysfunction, and hypoxia. Furthermore, recent advances to treat AKI with various LDNs in terms of 0D, 1D, and 2D nanomaterials have been discussed. Finally, the challenges and potential of LDNs for AKI treatment are highlighted.

## The pathogenesis of AKI

AKI has unclear, complex pathophysiology, which may be related mainly to oxidative stress, inflammation, mitochondrial dysfunction, and hypoxia.

### Oxidative stress

Oxidative stress is currently considered to be a critical factor in AKI, especially sepsis-related AKI [16]. In AKI patients, this oxidative stress may reflect increased autologous reactive oxygen species（ROS） production and hindered antioxidant capacity [17]. ROS is produced through multiple pathways, including mitochondrial electron transport mechanisms, the P450 system, monoamines, etc. ROS serves as a signal and regulates countless biological processes. It promotes the production of downstream pro-inflammatory cytokines by activating ROS-dependent phosphoinositol-3 kinase [18]. Regulation of HIF stability adapts to hypoxia [19] and promotes autophagosome formation by oxidative cysteine protease autophagy-associated gene-4 [20]. Oxidative stress can lead to destructive processes that harm cellular structures, such as cell membranes, lipids, proteins, lipoproteins, etc. [21–26]. For example, oxidative stress may damage proteins and cause them to undergo conformational modification, which leads to alteration or loss of their enzymatic activity [25, 27]. Excessive hydroxyl radicals can also cause lipid peroxidation, which damages the cell structure. Lipid peroxidation is a free radical chain reaction that diffuses very quickly and affects many lipid molecules [27]. DNA is also susceptible to oxidative stress-related damage, and it has been shown that oxidative stress can result in two mutations in 8-hydroxydeoxyguanosine (8-OHdG) [28]. Furthermore, it may also result in the loss of epigenetic information [29]. In addition, Valavanidis et al. [30] have proposed that 8-OHdG levels in tissues are biomarkers of oxidative stress.

ROS can also cause endothelial cell dysfunction by increasing vascular permeability and platelet adhesion [31]. Because blood vessels are susceptible to ROS generation, ROS can particularly easily affect oxygenation in the renal medulla [32]. A large number of superoxide molecules produced by leukocyte particles has been observed in patients with sepsis, which further enhances adhesion molecule activity and endothelial activation [33]. Excessive superoxide generation, accumulation, and inflammation can also directly lead to vascular structural damage, leakage, and tissue edema [34]. Therefore, ROS-mediated injury may be caused by changes in the local microcirculatory alterations and the increased oxygen demand for transport activity induced by oxidative stress.

### Inflammation

Inflammation plays an essential role in the pathogenesis of AKI. Endothelial cell injury, activation, and subsequent interaction with immune cells initiate the inflammatory cascade [35]. Renal ischemia–reperfusion (IR) injury leads to the destruction of the perivascular matrix, which significantly increases the permeability of the endothelial cell barrier. In ischemic AKI models, using matrix metalloproteinase-2 specific gene deletion therapy or minocycline, a broad-spectrum MMP inhibitor, improves microvascular permeability and renal injury [36, 37]. Therefore, endothelial barrier disruption may be caused by the activation of matrix metalloproteinase-2 or matrix metalloproteinase-9 [38]. Activated white blood cells bind to endothelial cells through these adhesion molecules and infiltrate into other sites. In animal studies, blocking or deleting these adhesion molecules has been shown to protect against kidney injury [39–41]. Activated leukocytes can cause additional endothelial cell damage and endothelial barrier permeability disorder [42].

Neutrophils are the earliest white blood cells that accumulate in the kidney after renal injury and are the major contributing factor to further renal injury after reperfusion by releasing ROS, proteases, elastase, and cationic peptides [43]. Neutrophils secrete chemokines and pro-inflammatory cytokines, forming a positive feedback pathway for neutrophil activation and recruitment and mediating renal injury through synergistic interaction with other white blood cells, including natural killer cells, monocytes, and macrophages [44, 45]. Stimulated neutrophils can produce superoxide anions, which are activated during adhesion. Superoxide is degraded into hydrogen peroxide by superoxide dismutase (H_2_O_2_). In addition, superoxide can be converted to HOCl, OH- or other ROS by myeloperoxidase (MPO) [46, 47]. TNF-α and IL-6 are pro-inflammatory factors released by congenital dendritic cells that initiate neutrophil recruitment and play an essential role in neutrophil recruitment, particularly in the kidney [48]. Macrophage infiltration increased significantly within an hour of Ischemia–reperfusion injury in AKI mice with ischemic kidney, peaked at 24 h, and lasted for 7 days [49]. After renal IR injury, macrophages (M1) infiltrate and activate by releasing ROS and pro-inflammatory cytokines such as TNF-α and IL-6. It can stimulate the activity of other white blood cells, thereby activating the Th1 immune response and inducing renal tissue damage [50].

### Mitochondrial dysfunction

Mitochondrial dysfunction is one of the critical conditions for the development of AKI [51, 52]. The primary source and target of intracellular ROS are mitochondria [53]. Mitochondria produce low ROS levels at their stroma sites, which act as signals and regulate many biological processes. However, in AKI, with the prolonging of cell hypoxia time, the metabolism of the intracellular electron transport chain changes, resulting in insufficient available oxygen to produce ATP, which increases electron leakage and increases mitochondrial ROS generation [54, 55].

In addition, elevated receptor-interacting protein kinase 3 (RIPK3) has been found in patients with septic AKI [56]. RIPK3 can inhibit mitochondrial complex I and III by increasing the expression of NADPH oxidase 4 and mitochondrial transposition and promoting mitochondrial dysfunction in the kidney [57, 58]. Therefore, mitochondrial damage may cause multiple organ dysfunction in sepsis patients [59, 60]. Down-regulation of the adaptive process of mitochondrial fission in AKI may further aggravate mitochondrial destruction, spreading ROS-induced damage in cells. Several in vitro studies have also revealed changes in mitochondrial shape, such as fewer cristae due to swelling of the cristae space and mitochondrial matrix and vacuolation of mitochondria [56, 61]. Mitochondrial removal can occur through two pathways: mitochondrial autophagy and apoptosis. Local and extensive mitochondrial damage leads to an overall decrease in the mitochondria's permeability.

Consequently, ROS and its products accumulate, and mitochondrial membrane channels are opened. Apoptotic bodies form due to the action of the apoptotic protease activator factor-1 protein, which initiates the downstream intrinsic apoptotic cascade mediated by procaspase-9 [62, 63]. Patients with septic AKI are prone to the destruction of mitochondria, which leads to further damage to antioxidant production [64] and an increase in the production of ROS.

In addition, the accumulation of ROS levels can lead to the upregulation of uncoupling protein-1 [65], which leads to excessive proton leakage, which damages ATP synthesis and simultaneously reduces the activity of cellular energy-dependent processes that may lead to cell death [66]. ATP depletion prevents Na^+^/Ca^2 +^ antiporter channels from pumping calcium out of cells, so calcium accumulates in cells [67]. In addition, calcium in the endoplasmic reticulum can lead to intracellular calcium overload through redistribution [68]. Increased cytoplasmic calcium activates calcium-dependent phospholipase A2, endonuclease, and protease within cells, causing apoptosis to begin [68, 69]. Thus, AKI causes mitochondrial damage, which results in a significant accumulation of ROS and Ca^2+^, oxidative stress, microvascular damage, and cell damage, ultimately leading to AKI.

### Hypoxia

Hypoxia occurs in AKI under various clinical and experimental conditions [70–72]. The cell's response to hypoxia centers on the hypoxia-inducible factor (HIF). HIF and hypoxia responses play an essential role in various types of AKI [73–75]. HIF-1α is a signal molecule ROS. Hypoxia leads to increased production of ROS in the electron transport chain, which may increase the stability and activity of HIF-1α [76, 77]. The increase in ROS prevents hydroxylation and degradation of HIF-1α and HIF-2α [78]. Erythropoietin is produced when HIF is activated [79, 80]. HIF increases the blood's hemoglobin level, thereby improving tissue hypoxia. However, excessive increased ROS can promote HIF-1α degradation through the ubiquitin proteasome system [81]. HIF can be a double-edged sword; although the cell protective mechanism driven by HIF can protect the kidney, it may fail when hypoxia worsens further, as it also occurs in AKI [82]. It has been reported that in the case of long-term hypoxia, the large production of HIF leads to the overproduction of vasoconstrictor proteins and ROS-inducing proteins (such as iNOS), as well as proteins that promote fiber formation [83, 84].

Renal medulla hypoxia is common in AKI Renal medullary hypoxia is an essential driver of AKI's transition to chronic kidney disease (CKD) and tendency to develop [85, 86]. The metabolic demand of the renal medulla is exceptionally high, and diffuse oxygen shunt may also exist in the renal medulla microcirculation, which may further affect the renal medulla oxygen supply [87]. Hypoxia in the renal medulla can lead to tubule damage, and Na^+^/K^+^-ATPase and transporters in tubule epithelial cells are incorrectly located, reducing the oxygen utilization efficiency of sodium reabsorption [88]. In addition, further aggravation of hypoxia leads to tubular cell death and may even lead to endothelial cell death to a certain extent.

## Low-dimensional nanomaterial applications in AKI treatment

**Fig.1:**
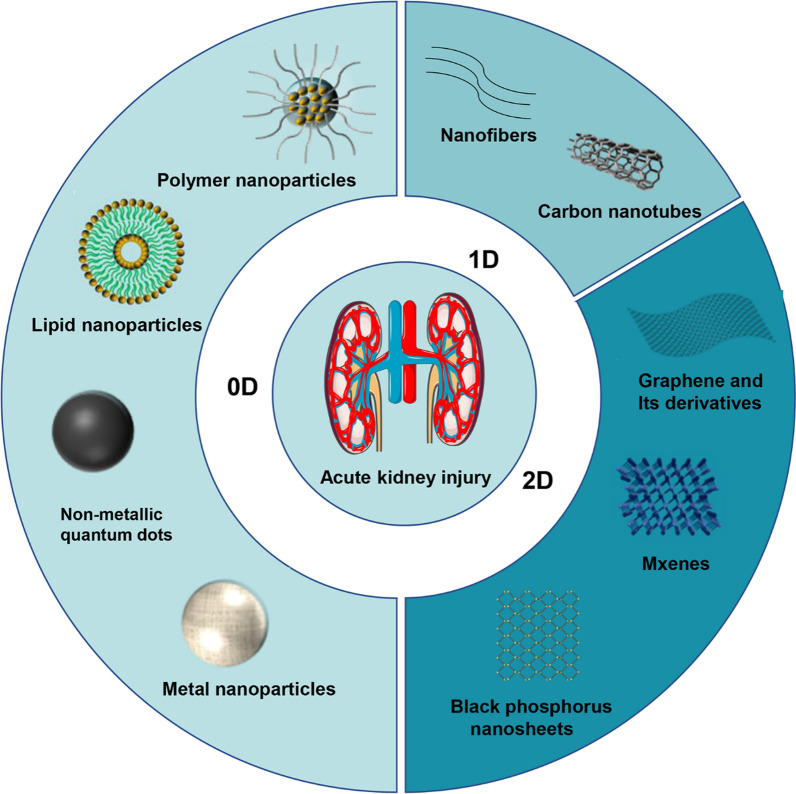
Illustration depieting different types of LDNs used for AKI treatment

In terms of treatment, the hospital can only carry out auxiliary treatments for AKI, such as fluid rehydration and kidney dialysis, and there is no other particular medication for the treatment of AKI. Consequently, the exploration of practical, minimally invasive, or non-toxic treatments for AKI remains an important objective. Nanomedicine based on biomaterials, especially low-dimensional nanomaterials (LDNs), has received much attention as an emerging strategy for treating AKI.（Fig. 1） According to their dimensions, LDNs can be generally divided into zero-dimensional (0D) materials, one-dimensional (1D) materials, two-dimensional (2D) materials. LDNs may be the biomaterials with the most favoring structural characteristics, such as small size, high solubility, strong reactivity, fewer adverse reactions, and a high drug loading rate [89, 90]. The following sections provide a summary of recent advances in the use of different types of LDNs to treat acute kidney injury (Table 1) .

### Zero-dimensional nanomaterials

In zero-dimensional (0D) nanomaterials, all three dimensions are confined to the nanometer scale (< 100 nm). Common 0D nanomaterials include quantum dots, nanospheres, liposomes, polymer nanoparticles (NPs), metal NPs, magnetic NPs, dendritic macromolecules, polymer micelles, solid lipid nanoparticles, etc. [91–96].

#### Metal-based nanoparticles

Metal-based NPs include metal NPs (e.g., iridium, silver, gold, platinum, zinc, and iron), metal oxide NPs (e.g., titanium dioxide, silver oxide, and zinc oxide), and magnetic NPs [97]. Generally, metal-based NPs have a small size, large surface area, and easy function modification. Most large NPs are not easily absorbed by the kidneys, resulting in potential organotoxicity. Due to this, the use of large-scale NPs in the treatment of AKIs is limited. The glomerulus can rapidly eliminate ultra-small NPs with a diameter below the renal filtration threshold (~ 10 nm) [98]. Metal-based NPs have good antioxidant activity and low biotoxicity, which can be excreted by the kidney and are potential therapeutic agents for the prevention of AKI.

**Fig. 2:**
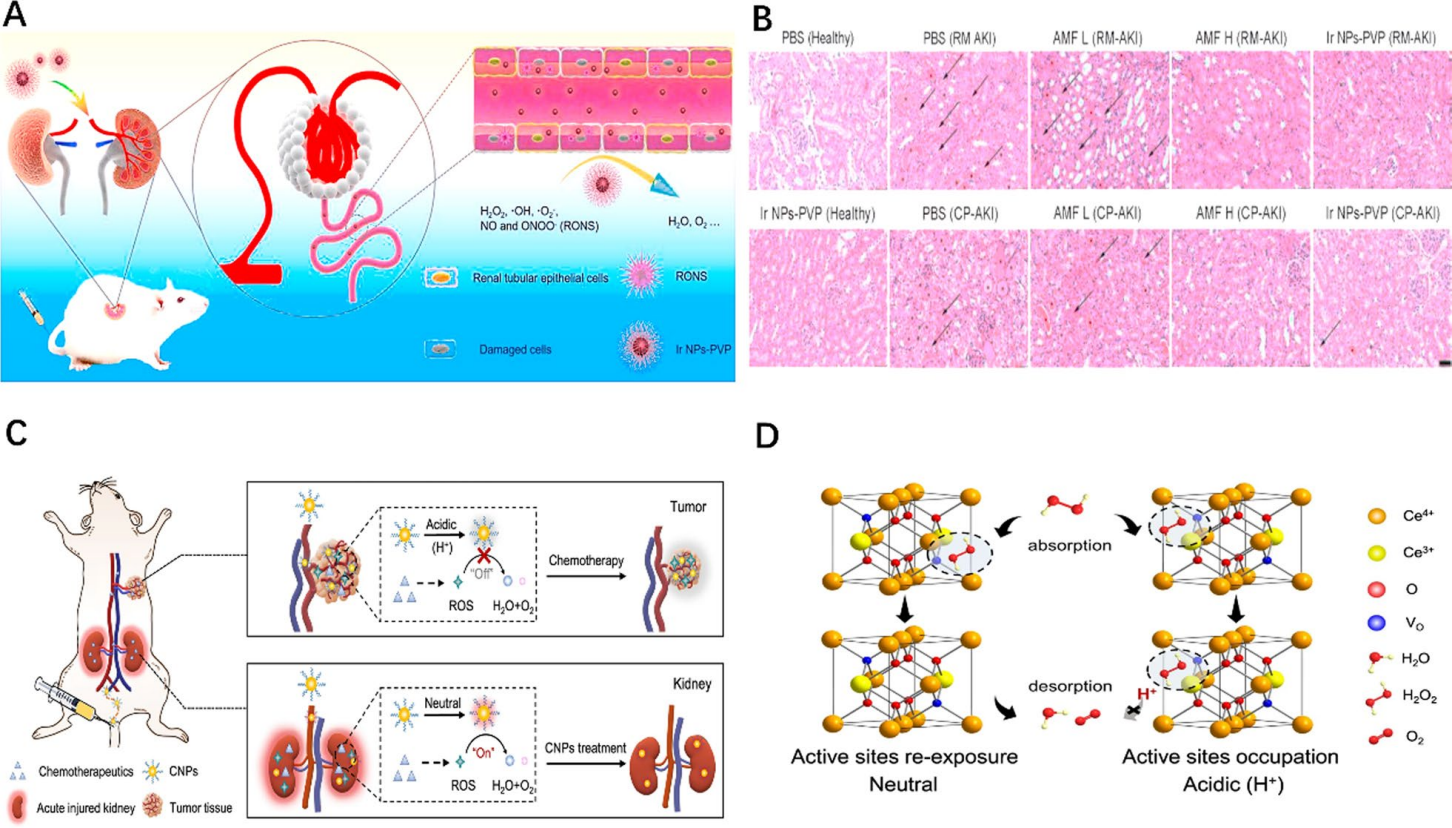
**A** Schematic illustration of Ir NPs-PVP as ROS scavengers and anti-oxidative drugs for AKI therapy. **B** H&E staining of renal tissues in different groups. Reprinted with permission from [99] **C** Schematic illustration of catalytic activity tunable CNPs for the treatment of AKI. **D** Chemical mechanism of CNPs modulating catalytic activity. Reprinted with permission from [100]

Iridium NPs have multienzyme activity and excellent scavenging ability for ROS, exhibiting great potential in AKI treatment. Zhang et al. [99] developed ultra-small polyvinylpyrrolidone-modified iridium nanoparticles (Ir NPs-PVP) as an effective nanoplatform for AKI treatment. They demonstrated that Ir NPs-PVP alleviated AKI by scavenging ROS. Concretely, Ir NPs-PVP could effectively improve the cell viability of H_2_O_2_-treated HEK293T cells and decrease the ROS level in a concentration-dependent manner. Additionally, an in vivo biodistribution study revealed that the ultra-small Ir NPs-PVP rapidly accumulated in the kidney and exhibited a higher renal enrichment than those in healthy mice. Moreover, these Ir NPs-PVP could be quickly excreted through the kidney and urine, leading to a significantly lower systemic toxicity in vivo. More importantly, after treatment with Ir NPs-PVP for one month, no significant damage and inflammatory changes were observed in the renal tissues of AKI mice, suggesting the promising therapeutic potential of Ir NPs-PVP (Fig. 2A and B).

Ceria nanoparticles (CNPs) is another type of metal NPs with promising potential for treating AKI. Weng et al. [100] developed catalytically active tunable CNPs for AKI treatment. CNPs were very active in the decomposition of H_2_O_2_ in neutral conditions but inert in acidic conditions. The mechanism may be the redox reaction between CNPs and H_2_O_2_ (H_2_O_2_ + 2Ce^4+^ → O_2_ + 2H^+^ + 2Ce^3+^ + Vo) adsorbed on the surface under neutral conditions. However, excessive H^+^ can effectively reduce the conversion of Ce^4+^ to Ce^3+^, thus destroying the re-exposure of the active catalytic site and thus blocking the antioxidant cycle (Fig. 2C and D). The protective effects of CNPs on HK-2 and ES-2 cells have been reported. Similar to the previous results, CNPs significantly reduced cytotoxicity in a neutral environment but not in an acidic environment. In vivo, CNPs did not interfere with the efficacy of chemotherapy drugs due to their specific inhibition of antioxidant activity in an acidic tumor microenvironment. Additionally, the authors found that CNPs activated the Nrf2/Keap1 signaling pathway to restore redox homeostasis in renal cells, thereby preventing renal cell apoptosis in AKI mice.

In another study, Yu et al. [101] developed a ROS-responsive and mitochondria-targeted nano-delivery system based on CNPs for AKI treatment. Specifically, triphenylphosphine (TPP)-modified CNPs were coated with a ROS-responsive polymer (mPEG-TK-PLGA) and simultaneously loaded with atorvastatin to form Atv/PTP-TCeria NPs. The results showed that the generated NPs not only exhibited good antioxidant and anti-apoptotic activities in vitro, but also effectively reduced oxidative stress and inflammation in septic-induced AKI mice, and protected the cellular mitochondrial structure, thereby reducing kidney damage. This nano system overcomes the disadvantage that CNPs cannot selectively target mitochondria, and super-small CNPs is easy to agglomerate, thus improving therapeutic efficiency.

**Fig.3:**
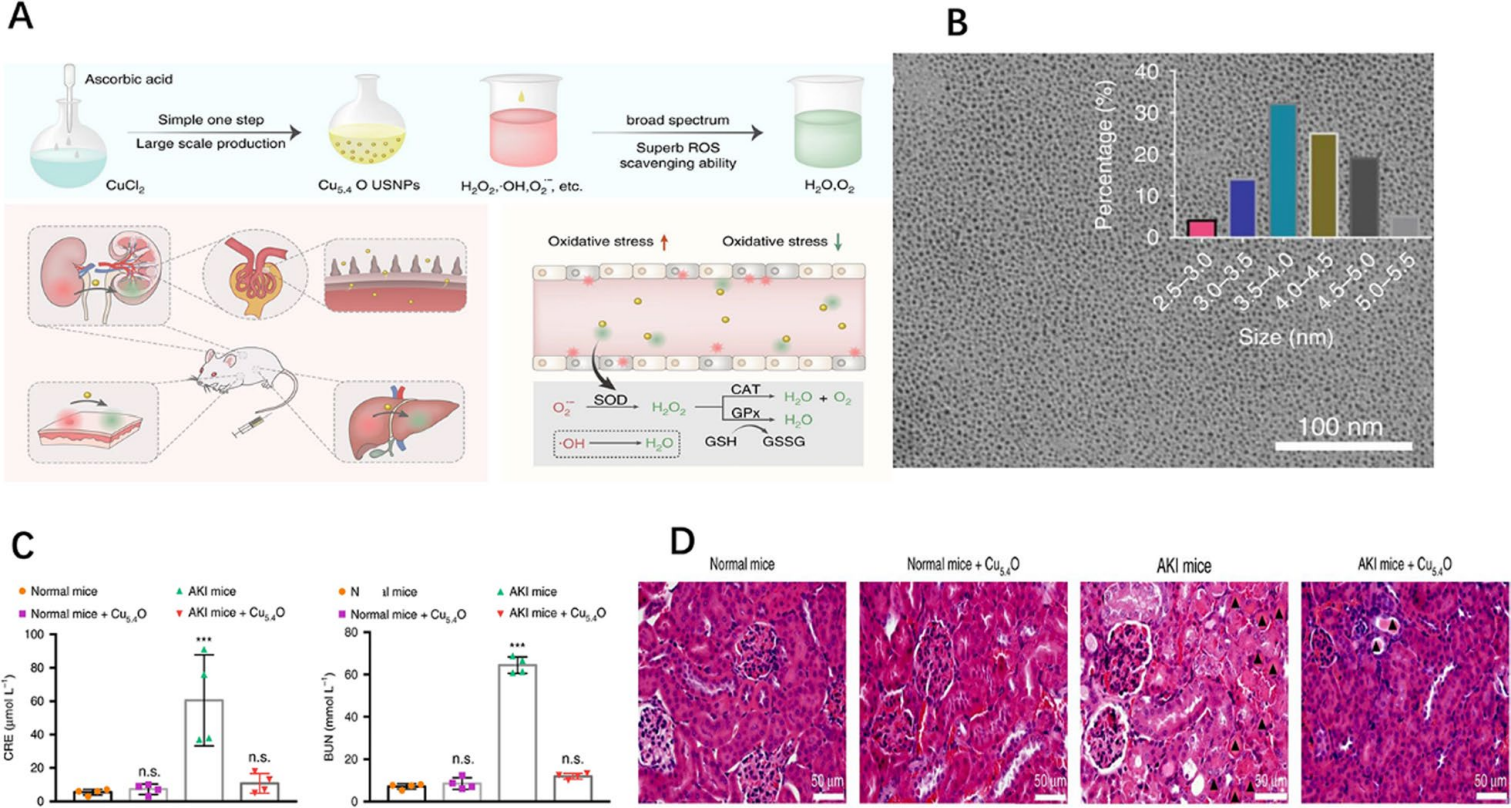
**A** Schematic illustration of Cu_5.4_O USNPs as broad-spectrum ROS scavenging drugs for AKI treatment. **B** TEM image of Cu_5.4_O USNPs. **C** Blood biochemical levels of AKI mice after different treatments. **D** H&E staining of renal tissues after different treatments. Reprinted with permission from [106]

Cu is an essential element for the human body. Previous studies have also shown that Cu-based nanomaterials can be used to scavenge ROS [102, 103]. Cuprous oxide (Cu_2_O) NPs have good catalytic activity and can promote electron transfer reaction passivation of H_2_O_2_ or OH· by simulating peroxidase [104, 105]. Therefore, Liu et al. [106] believed that combining Cu_2_O and Cu nanocrystals could simultaneously obtain broad-spectrum enzyme catalytic performance and antioxidant activity. The efficient separation of electron holes between Cu_2_O and Cu improved the overall ROS scavenging ability of Cu NPs [107, 108]. They successfully prepared ultra-small Cu_5.4_O nanoparticles (Cu_5.4_O USNPs), which has significant antioxidant efficiency. In addition, animal experiments showed that Cu_5.4_O USNPs had a high renal clearance rate without substantial toxicity, and their renal therapeutic effect was excellent (Fig. 3A–D).

In addition to the above metals, LDNs based on other metals also hold advantages in AKI treatment. For example, Liu’s group developed ultra-small rubidium oxide nanoparticles (RuO_2_ NPs) for treating AKI [109]. RuO_2_ NPs could mimic catalase, peroxidase, and other enzymes in catalytic performance and show good antioxidant activity, thus they have great potential in reducing ROS-induced apoptosis. In addition, RuO2 NPs could be excreted through the glomerulus and showed effective renal storage, renal clearance, and long-term biosafety in vivo. The excellent performance of these ultra-small RuO_2_ NPs highlighted their promise as a multi-enzymatic nanoenzyme for AKI prevention. In Ni’s study, [110] molybdenum (Mo)-based polyoxometalate clusters (POM) served as a novel nano-antioxidant that could be preferentially absorbed in the kidney, protecting it from damage. These POM nanoclusters have variable valence molybdenum ions, which could remove harmful ROS. In vivo POM nanoclusters showed an enhanced accumulation in the kidney and effectively improved the symptoms in AKI mice, demonstrating their potential as an intelligent, adaptive nano-therapy for AKI. Additionally, oral administration of pure gold (Au) NPs protected against acetaminophen-induced nephrotoxicity, and Au NPs treatment restored adenosine triphosphate (ATP) enzyme and glucose-6-phosphatase activities to normal levels [111]. Au NPs can also be used as nanocarriers to deliver fig leaf extracts for the treatment of AKI induced by enoxaparin [112].

#### Non-metallic quantum dots

The development of inorganic, non-metallic nanomaterials with ROS scavenging properties, such as carbon-based nanomaterials and graphene quantum dots, opens up a new avenue for treating various ROS-related diseases [101, 115].

**Fig. 4:**
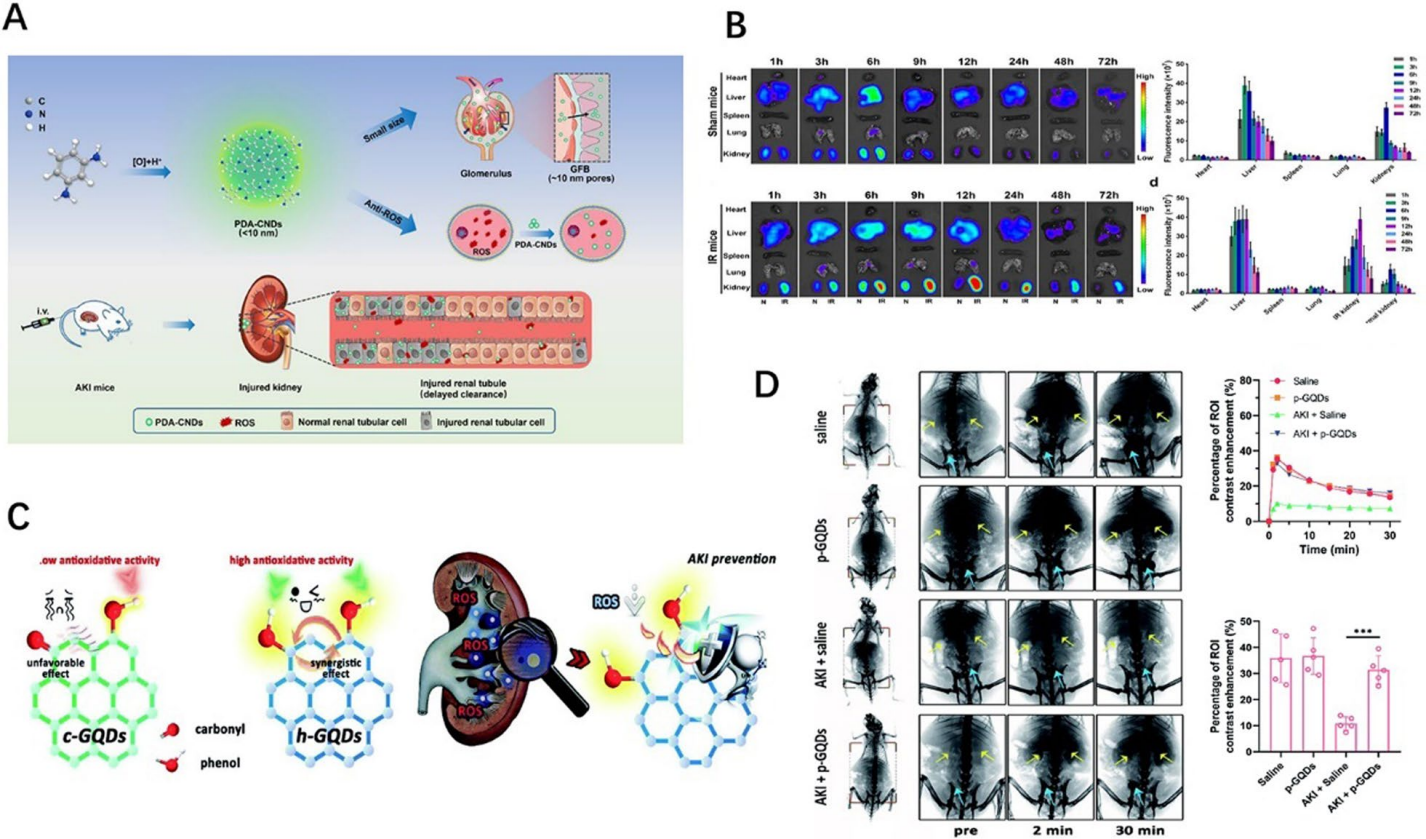
**A** Schematic treatment of AKI using PDA-CNDs. **B** Biological distribution of PDA-CNDs in each group of mice. Reprinted with permission from [113]. **C** Schematic illustration of antioxidative activity of h-GQDs and their usage as ROS scavengers for treating AKI. **D** X-ray images of each group after intravenous injection of contrast agent. Time-dependent ROI analysis of X-ray intensity and density variation in the kidneys of mice after various treatments. Reprinted with permission from [114]

Carbon nanodots (CNDs) are a new type of carbon-based nanomaterial, usually smaller than 8 nm, with excellent stability and biocompatibility. They have been widely used in catalysis, targeted drug delivery, and other biomedical fields. In addition, different functional groups can easily modify the CND surface, which provides a broader prospect for regulating its physicochemical properties. Some studies have shown that some CNDs can scavenge free radicals in vitro [116, 117]. Therefore, Gao et al. [113] evaluated the antioxidant capacity of phenylenediamine-based CNDs (PDA-CNDs) and their potential for treating AKI. PDA-CNDs were discovered to accumulate primarily in the kidneys of IR-AKI mice. Compared with normal mice, PDA-CNDs were preferentially aggregated and retained in IR-induced AKI kidneys (Fig. 4A and B). Increased PDA-CND absorption by HK2 cells, increased AKI-related microvascular permeability allowing easy efflux of systemically administered NPs to the site of injury, and renal damage slowing PDA-CND clearance may all play a role in the more significant and better absorption and more prolonged PDA-CND clearance. Due to this, PDA-CNDs accumulate slowly in renal tubules. In vivo experiments demonstrated the role of PDA-CNDs as ROS scavengers in alleviating oxidative stress-induced renal injury by reducing the production of superoxide and pro-inflammatory factors. Therefore, PDA-CNDs may be a powerful drug treatment approach for AKI.

Heteroatomic doping is a powerful method to regulate the fluorescence backdrops of carbon quantum dots (CQDs). Elemental doping in CQD has effectively designed its intrinsic properties [118]. Due to its special electronegativity, selenium (Se) is an essential trace element with unique chemical properties, such as redox reactions. It plays a significant role as an antioxidant by incorporating selenoproteins [119]. In one study, Se-doped carbon dots were hydrothermally treated with se-cysteine to produce simple, high-yield green fluorescent carbon-selenium quantum dots (SeCQDs) [120]. SeCQDs exhibited comprehensive ROS scavenging properties, including H_2_O_2_, O_2_^−^, and ·OH. In AKI mice, SeCQDs accumulated rapidly and almost entirely in the kidney. More importantly, treatment with SeCQDs significantly improved the renal injury of AKI mice.

Graphene quantum dots (GQDs) are another type representative non-metallic quantum dots used in AKI treatment. Wang et al. [114] developed phenol-like group functionalized GQDs (h-GQDs) with excellent ROS scavenging ability and kidney specificity for AKI antioxidant treatment. The abundant phenolic groups on h-GQDs are effective ingredients with antioxidant effects, similar to natural polyphenols. Their study showed that synergies between adjacent phenolic groups of h-GQDs and eliminating unfavorable carbonyl compounds from h-GQDs enhanced antioxidant capacity. Moreover, their found that PEGylated h-GQDs (p-GQDs) were more likely to accumulate in injured kidneys and showed prolonged renal retention. P-GQDs treated AKI mice had significant recovery of renal function (Fig. 4C and D). Subsequent experiments demonstrated that h-GQDs completely protected the kidney from oxidative damage in AKI mice at only low doses, with no evidence of toxicity.

#### Lipid nanoparticles

According to the structure and composition, lipid nanoparticles, can be divided into solid lipid nanoparticles (SLNs), nano-structured lipid carriers, lipid-drug conjugates and lipid-polymer hybrid nanoparticles.

SLNs are mainly made of phospholipids and solid lipids. It has the advantage of using physiological lipids, avoiding the use of organic solvents in the preparation process, and is generally considered safe, biocompatible and biodegradable [121–123]. They can be a good alternative to polymer systems due to their lower toxicity, ability to protect active pharmaceutical ingredients from degradation. It is more attractive to functionalize them with ligands to accomplish kidney targets [124, 125]. Therefore, SLNs modified by ligands may be a targeted delivery system that prolongs drug release. In one study, Hu et al. [126] prepared sialic acid (SA)-conjugated SLNs (SA-NPs) loaded with dexamethasone (DXM). DXM is widely used in treating AKI due to its anti-inflammatory and antioxidant abilities, but it also has specific toxicity. Therefore, they chose to seal DXM in SLNs and target its release into the kidney with inflammation, thereby reducing its systemic toxicity. SA was selected as a ligand to target inflammatory vascular endothelial cells. SA-NPs could be specifically internalized by inflammatory vascular endothelial cells, and the mechanism may be related to the specific binding of SA to the E-selectin receptor expressed by inflammatory vascular endothelial cells. In vivo biological distribution results revealed that SA-NPs exhibited increased accumulation in renal tissues and significantly elevated DXM content in the kidney of AKI mice, further suggesting the renal specificity of SA-NPs. Further studies showed that SA-NPs could effectively improve biochemical blood indexes such as creatinine in AKI mice and reduce oxidative stress and pro-inflammatory cytokines, which was further confirmed by histopathological changes.

Similarly, Liu et al. [127] reported that sialic acid-modified lipid calcium phosphate nanoparticles (SA-NPs). The SA-NPs not only improved the drug loading efficacy of DXM but also served as inflammatory-specific targeting nano system that could be more efficiently internalized into cells through E-selectin receptor-mediated endocytosis. They found that pretreatment with free SA could block the combination of SA-NPs with relevant receptors on the cell surface, further suggesting the role of modified SA in prompting the cellular uptake. In addition, pharmacological studies showed that SA-NPs loaded with DXM significantly increased their residence time in vivo, and their plasma half-life was 1.7 times higher than that of free DXM. Accordingly, compared with free DXM, SA-NPs performed better in ameliorating kidney damage.

In addition to small-molecule drugs, lipid NPs can also efficiently deliver biomolecules. MicroRNAs (miRNAs) participate in many critical biological processes, including apoptosis, cell proliferation, differentiation, biological characteristics, and physiological functions [128]. MiRNAs can also be loaded into liposome for AKI treatment. Zhang et al. [129] considered miR-500a-3p an appropriate therapeutic miRNA for AKI and loaded it into liposome, with the aim to improve its therapeutic efficacy against AKI. The results suggest that miR -500a-3p-loaded liposomes (miR-LIP) directly controlled the expression of receptor interacting protein kinas 3 (RIPK3) and mixed lineage kinase domain-like protein (MLKL), a main regulator for necrosis and apoptosis, thereby reducing the severity of kidney injury. In vitro experiments showed that miR-LIP significantly regulated the phosphorylation of MLKL and RIPK3 and reduced inflammatory responses. Further, western blot analysis showed miR-LIP decreased phosphorylation of NF-κB, which might contribute to inhibiting the inflammation in the kidney cells.

#### Polymer nanoparticles

Polymer NPs are classified as natural or synthetic polymers modulated by different monomers or prefabricated polymers and adjusted by various parts to precisely modulate the performance of the polymer NPs. Therapeutics based on polymer NPs show great promise in treating multiple diseases because their structures can be flexibly modified, giving them better biocompatibility, bioavailability, enhanced permeability, and better retention time [132–134].

**Fig. 5:**
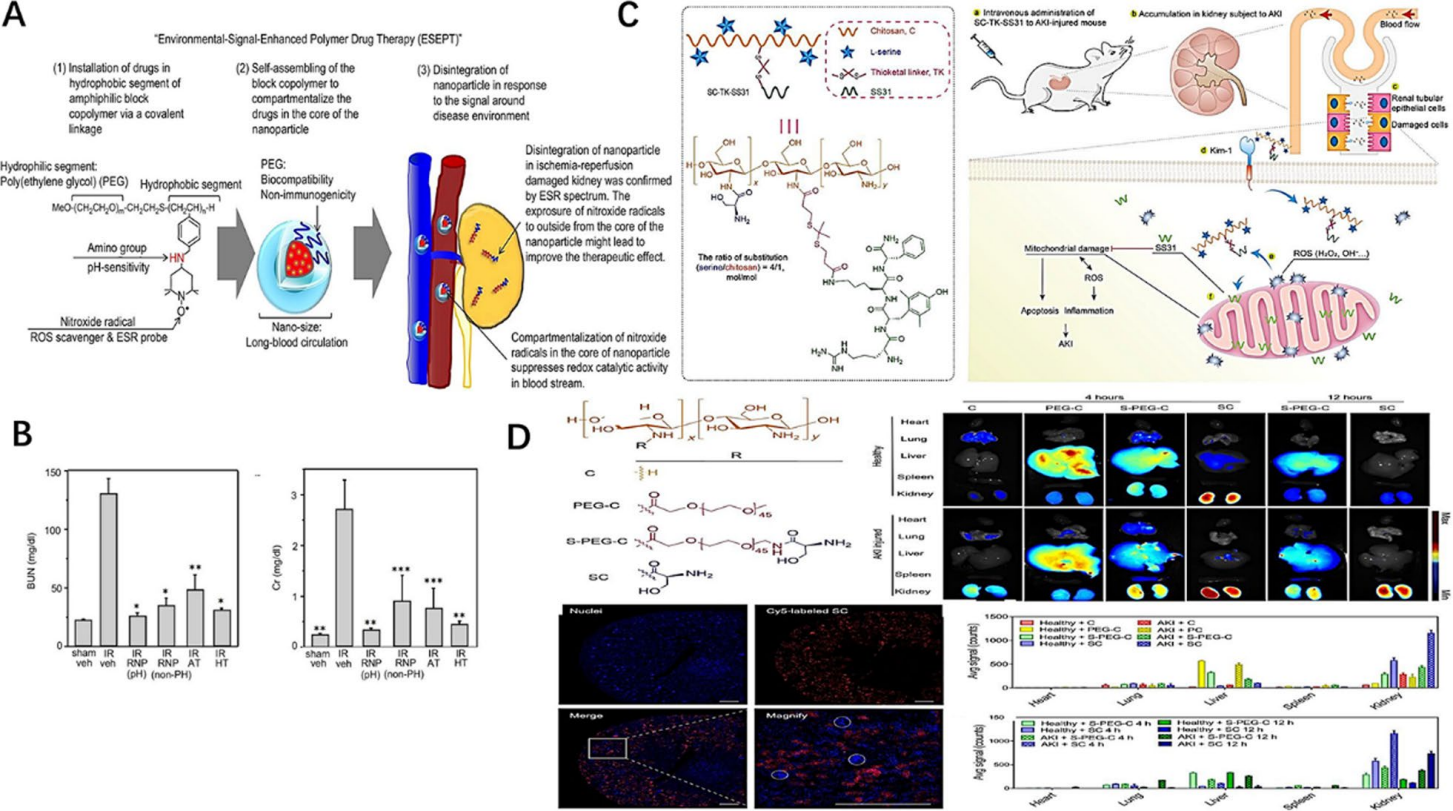
**A** Schematic of “Environmental-Signal-Enhanced Polymer Drug Therapy (ESEPT)”. **B** BUN and sCr levels at 24 h after reperfusion following 50 min ischemia. Reprinted with permission from [130]. **C** Chemical structure of SC-TK-SS31 and schematic diagram of SC-TK-SS31 in the treatment of AKI. **D** Renal distribution and renal tubules accumulation. Reprinted with permission from [131]

Yoshitomi et al. [130] proposed “Environmental Signal-Enhanced Polymer Drug Therapy” (ESEPT) for AKI (Fig. 5A). They used a poly(ethylene glycol)-*b*-poly(methylstyrene) (PEG-*b*-PMS) block copolymer to link to 2,2,6,6-tetramethylpiperidine-N-oxyl (TEMPO) through an amine linkage (PEG-*b*-PMNT). PEG-*b*-PMNTs self-assembled into polymer micelles with core–shell structure in aqueous media, which decomposed under acidic conditions due to the protonation of amino groups located at the core of nitroxide-radical-containing nanoparticle (RNP^pH^). Meanwhile, RNP^pH^ can catalyze ROS scavenging. The site of nephropathy is in an acidic environment where RNP^pH^ can be broken down to remove ROS. The authors confirmed the disintegration of RNP^pH^ in the damaged kidney. RNP^pH^ was superior to low molecular weight TEMPO derivative in alleviating renal dysfunction. In addition, the outcome of RNP^pH^ in reducing sCr and blood urea nitrogen (BUN) was much greater than that of nanoparticles without pH-responsive ability, suggesting that the disintegration of RNP^pH^ might enhance its treatment effect (Fig. 5B). RNP^pH^ also had inhibitory effects on superoxide anion production, the inflammatory cytokine IL-6, and lipid oxidation, further ameliorating AKI.

Liu et al. [131] developed a ROS-responsive prodrug (SC-TK-SS31) to achieve effective AKI treatment (Fig. 5C). SS31 is a mitochondria-targeted peptide with antioxidant activity that can be used for treating AKI. SC-TK-SS31 was produced by conjugating SS31 to L-serine-modified chitosan (SC). Chitosan bound to heparin sulfate and glucosamine sulfate in the glomerulus via electrostatic interaction, allowing active renal targeting and then effectively reaching the renal tubules. Based on the characteristics of kidney targeting, SC could be deeply ingested into the injured kidneys. But in healthy mice, SC was rapidly cleared from the kidneys (Fig. 5D). Also, L-serine binds to the transmembrane protein KIM-1, which is overexpressed in renal tubules. Thus, SC-TK-SS31 can be enriched, retained, and internalized in damaged kidneys. Triggered by ROS, SS31 was released in damaged renal tubular epithelial cells. SC-TK-SS31 was further verified to significantly improve the therapeutic effect of SS31 on AKI through in vitro and in vivo experiments.

In another study, Liu et al. [135] also designed pH-responsive nanoparticles for renal targeted delivery of SS31 for AKI. It has been found that CD44 receptors are increased in damaged kidneys and that hyaluronic acid can target CD44 [136–138]. Thus, hyaluronic acid can be used for targeted delivery to injured kidneys. SS-31 were encapsulated into nanopolyplexes for improving its in vivo biodistribution and delivery efficiency. The apparent charge of anionic hyaluronic acid and cationic chitosan are susceptible to pH conditions and thus affect the electrostatic interactions of NPs. SS-31 was rapidly released under acidic pH conditions. Due to electrostatic imbalance, SS31 could be released in lysosomes at low pH, further targeting mitochondria, exerting anti-oxidation effect, and alleviating AKI. In vivo studies demonstrated that the nanopolyplexes showed better therapeutic effects.

Moreover, Hu et al. [139] prepared sialic acid-PEG-dexamethasone conjugate (SA-PEG-DXM) through esterification reaction, and simultaneously encapsulated DXM to form SA-PEG-DXM/DXM. More SA-PEG-DXM was assembled in the AKI kidney than in the non-SA-modified PEG-DXM, possibly due to the link between overexpressed E-selectin receptors and SA. SA in the SA-PEG-DXM conjugate significantly reduced lipopolysaccharide (LPS)-induced proinflammatory cytokine production by inhibiting Beclin-1/ATG5-ATG12-mediated autophagy. Compared with free DXM, SA-PEG-DXM/DXM micelles showed superior therapeutic efficacy in LPS-induced AKI mouse model, including improved renal function and inhibition of pro-inflammatory cytokines.

Poly (lactic-co-glycolic acid) (PLGA) NPs have high accumulation and passive targeting of renal tubules in IR kidneys [140]. Yu et al. [141] designed Oltipraz-loaded PLGA NPs (PLGA-Oltipraz NPs) to treat IR-induced AKI. PLGA-Oltipraz NPs could target the IR kidney more effectively at the initial stage. Meanwhile, the NPs could improve renal function and effectively protect the AKI kidney from inflammatory injury and collagen deposition. Specifically, PLGA-Oltipraz NPs could be selectively transported into renal tubular epithelial cells, and activated the expression of antioxidant stress-related Nrf2 and its downstream targets NQO1, GCLC, and Gpx2, thereby reducing renal injury and fibrosis. Thus, PLGA-oltipraz NPs hold great potential in IR-induced AKI treatment due to their renal target, anti-inflammatory properties, and great potential in clinical transformation.

Ureteral obstruction significantly increases COX-2 expression [142–144], and selective COX-2 inhibitors have been shown to reduce renal injury and apoptosis in a unilateral ureteral obstruction (UUO) mouse model of unilateral ureteral obstruction [145]. However, the side effects of selective COX-2 inhibitors hinder their clinical application. Yang et al. [146] utilized chitosan/siRNA NPs to alleviate renal injury via specific COX-2 knockdown in the UUO-induced AKI mouse model. Chitosan/siRNA NPs were found to accumulate in obstructed renal macrophages. Injection of COX-2 chitosan/siRNA NPs effectively reduced COX-2 expression and ameliorating tubule damage in UUO mice. In addition, COX-2 siRNA decreased the expression of TNF-α, IL-6, heme oxygenase-1 and cleaved caspase-3 in UUO mice. Suggesting that COX-2 siRNA could play an anti-oxidative stress and anti-apoptosis role. Chitosan/siRNA NPs have three advantages in clinical application. First, after intraperitoneal injection, the injured kidney is the main site of siRNA distribution. Second, chitosan/siRNA nanoparticles are delivered through macrophages, minimizing exposure to other organs. The last, extremely low doses of COX-2 siRNA were injected to reduce renal injury by reducing oxidative stress, inflammation, and apoptosis .

**Table 1:** An overview of different LDNs used in the treatment of AKI

Type	Nanomaterials	Animal model	Mechanism	Refs.
0D	Ir NPs-PVP	RM-induced AKI model; CP-induced AKI model	Scavenge RONS	[99]
0D	Ceria NPs	CP-induced AKI model; IR-induced AKI model; cyclophosphamide -induced AKI model	Scavenge ROS; regulate the ROS-involved genes by activating the Nrf2/Keap1 signaling pathway	[100]
0D	Atv/PTP-TCeria NPs	LPS-induced AKI model	Scavenge ROS; inhibit inflammatory response; protect mitochondrial structure	[101]
0D	Cu_5.4_O USNPs	CP-induced AKI model	Scavenge ROS	[106]
0D	FGP nanodots	RM-induced AKI model, CP-induced AKI model	Scavenge ROS	[147]
0D	Ultrasmall RuO_2_ NPs	RM-induced AKI model, CP-induced AKI model	Scavenge ROS; inhibit apoptosis	[109]
0D	Mo-based POM nanoclusters	CP-induced AKI model	Scavenge ROS by switching between reduced and oxidized forms	[110]
0D	PDA-CNDs	IR-induced AKI model; CP-induced AKI model	Scavenge ROS	[113]
0D	Functionalized GQDs	Glycerol-induced AKI mice	Possess broad-spectrum ROS-scavenging property	[114]
0D	Se-Doped CQDs	RM-induced AKI model, CP-induced AKI model	Possess broad-spectrum ROS-scavenging property	[120]
0D	Au NPs	Acetaminophen-induced nephrotoxicity??	Antioxidative, anti-inflammatory, and anti-angiogenic capabilities	[111]
0D	DXM-loaded SA-NPs	IR-induced AKI model	Specific recognition by E-selectin receptor; inhibit inflammatory response; inhibit apoptosis	[126]
0D	DXM-loaded SA-NPs	IR-induced AKI model	Reduce pro-inflammatory cytokines levels, oxidative stress, and cell apoptosis	[127]
0D	RNP^pH^	IR- induced AKI model	Scavenge ROS; pH-responsive	[130]
0D	SC-TK-SS31	IR-induced AKI model	ROS-triggered drug release to protect mitochondria from damage and reduce oxidative stress, inflammation, and cell apoptosis	[131]
0D	SS31-loaded nanopoly -plexes	LPS-induced AKI model	Inhibit oxidative stress and inflammatory response; protect mitochondrial structure	[135]
0D	DXM-loaded polymer micelles	LPS-induced AKI model	Mitigate generation of pro-inflammatory cytokines through inhibiting Beclin-1/Atg5-Atg12-mediated autophagy	[139]
0D	Oltipraz-PLGA NPs	IR-induced AKI model	Renal target, anti-inflammatory, and antioxidant	[141]
0D	Chitosan/siRNA NPs	UUO renal inflammatory model	Reduce oxidative stress, inflammation, and cell apoptosis; specifically knock down COX-2 in macrophages	[146]
1D	fCNT/siRNA	CP-induced AKI model	Enhance siRNA delivery to tubule cells	[115]
1D	SFP/BA NFs	CP-induced AKI mouse model	Scavenge ROS;the activation of cGAS/TING	[148]
2D	BPNSs	Glycerol-induced AKI model	Broad-spectrum ROS-scavenging property	[149]
2D	Ti3C2-PVP nanosheets	Glycerol-induced AKI model	Scavenge ROS; suppress oxidative stress-induced inflammatory response through inhibition of NF-κB signal pathway	[150]
2D	GO	CP-induced AKI model	Increase uptake of MSCs, growth factors secreted by MSCs, and essential factors in the blood	[151]
2D	GO-BSA	Endotoxin-induced AKI model	Adsorb ECM proteins and encourage their exchange to the intense renal damage tissue and expand its repair speed	[152]

### One-dimensional nanomaterials

One-dimensional (1D) nanomaterials refer to materials with one of the three dimensions between 0.1 and 100 nm in size [153]. The chemical, physical, electronic, and photoelectrical properties of 1D nanomaterials have been extensively studied, and a series of nanodevices, such as fiber lasers, electrodes, and light absorbers, have been constructed [154–156]. Interestingly, the size and dimensions of 1D nanomaterials allow them to efficiently encapsulate drugs for diseases treatment.

#### Carbon nanotubes

Carbon nanotubes, are one of the most representative 1D nanomaterials. Carbon nanotubes have been studied as delivery platforms for small interfering RNAs (siRNAs) [157, 158]. Ammonium functionalized single-walled carbon nanotubes (fCNTs) are unique fibrous macromolecules that can carry drugs [159]. FCNTs have excellent glomerular filtration and elimination properties [160–162]. The filtered portion of fCNTs is recollected at the brush edge of the proximal tubular cell (PTC) [161]. For the treatment of renal-related diseases, fCNTs can carry non-covalently bound siRNA to key physiological chambers of the kidney.

**Fig. 6:**
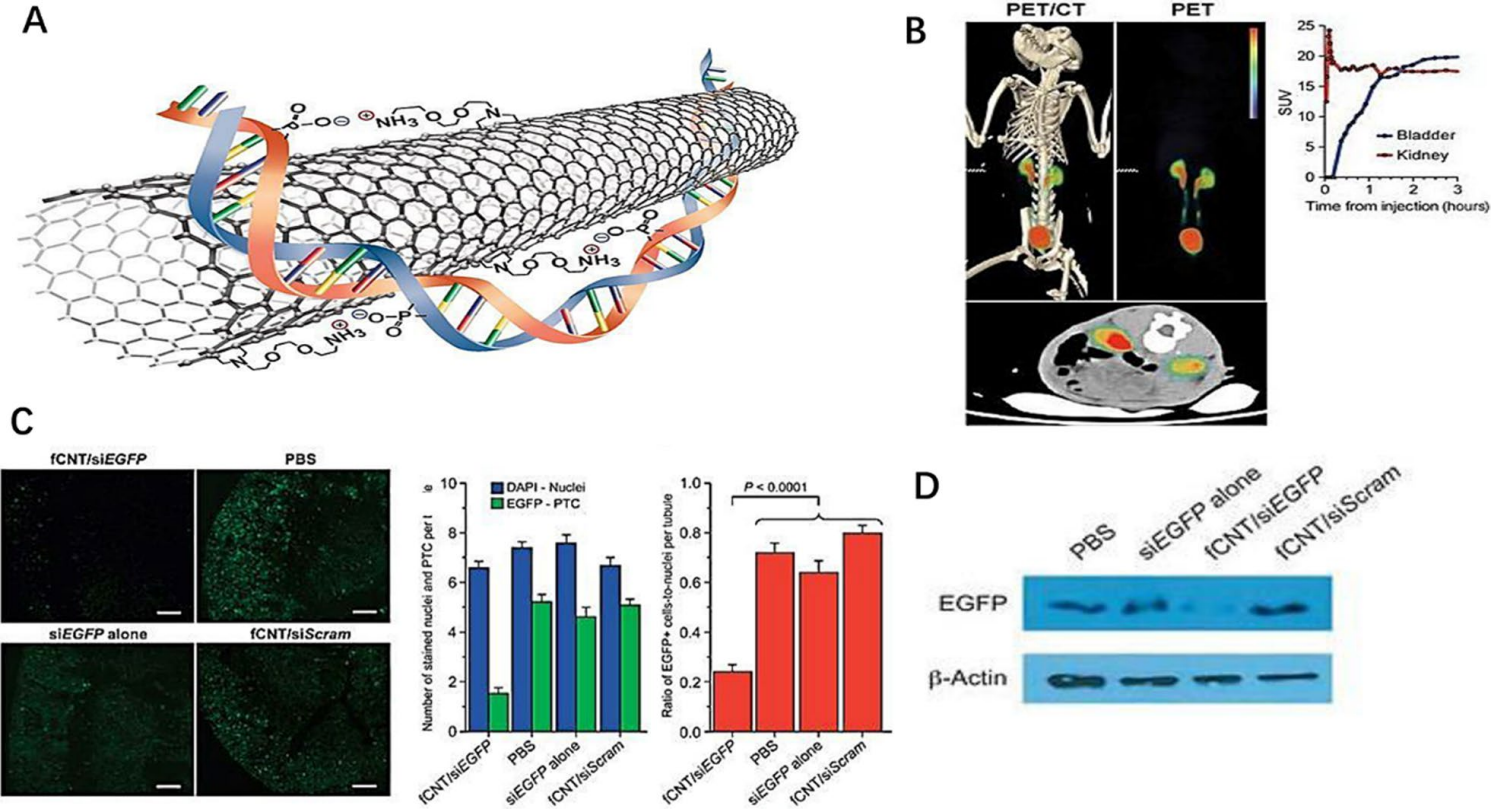
**(A)** Schematic diagram of carbon nanotube siRNA assembly. **B** PET/CT image of a representative 5-kg cynomolgus monkey; quantitative analysis of the standard uptake value in the bladder and kidney. **C** Frozen kidney section images of EGFP transgenic mouse models after treatment in different treatment groups; Quantification analysis of EGFP-positive cells; Ratio of EGFP-positive cells to total cells in the PTC as a standard of treatment effect. **D** Western blot analysis of renal cortex tissues of each treatment group. Reprinted with permission from [115]

Alidori et al. [115] used fCNTs to selectively and efficiently transport siRNAs into proximal renal tubule cells of cisplatin-induced AKI mice (Fig. 6A–D). Each fCNT could load up to four siRNAs. The pharmacokinetic results confirmed specific targeting of fCNT/siRNA in the kidney. Moreover, fCNTs showed excellent blood clearance, biological tissue distribution, and kidney elimination in monkeys. In vitro experiment, fCNT-mediated siEGFP reduced fluorescence expression by 92%, while the control siEGFP alone achieved a maximum inhibition of about 40% In animal studies, fCNT and EGFP-targeting siRNA were used to knock down tubule-specific genes. They found that renal cortical fluorescence was significantly reduced by 75% after treatment with fCNT/siEGFP, suggesting the effect of fCNT/siRNA in knocking down gene expression. Meprin-1β and p53 proteins play critical roles in depolarization and apoptosis of renal injury, respectively. Therefore, they subsequently explored whether siRNA targeting Trp53 and MEP1B could be delivered explicitly to proximal renal tubule cells using the fCNT platform. Expectedly, fCNT/siTrp53/siMep1b successfully reduced renal mRNA and protein expression after toxic renal injury. It also reduced fibrosis and immune cell infiltration. In summary, fCNT is a promising nanomedicine tool for powerful preventive treatments to mitigate AKI.

#### Nanofibers

As a type of 1D nanomaterial, nanofibers are well-known for their high surface-to-volume ratio and controllable pore structures. Nanofibers have been widely used for drug delivery. Liu et al. [148] utilized silk fibroin peptide (SFP) to fabricate nanofibers and further encapsulated baicalin (SFP/BA NFs) for AKI treatment. SFP/BA NFs significantly increased the water solubility and antioxidant activity of BA in vitro. The results of in vitro experiments showed that SFP/BA NFs could inhibit the ROS accumulation and mitochondrial membrane potential destruction induced by cisplatin. In vivo experiments showed that SFP/BA NFs could significantly improve cisplatin-induced renal injury.

### Two-dimensional nanomaterials

Two-dimensional (2D) nanomaterials refer to materials with two of the three dimensions between 0.1 and 100 nm in size [163]. Nanoplates, nanosheets, and nanowalls usually represent their forms. 2D nanomaterials include black phosphorus nanosheets (BPNSs), MXenes, graphene and its derivatives, etc. The high surface volume ratio, unsaturated site coordination rate, biocompatibility, and degradability of 2D nanomaterials make them widely used in energy, environment, electronics, optoelectronics, and biomedical fields [164–166]. 2D nanomaterials, as novel nanomaterials with an ultrathin layer structure topology, are attracting more and more attention in biomedical applications due to their outstanding physical and chemical properties, which can absorb many drug molecules for disease treatment. To our knowledge, the literature on 2D nanomaterials for cancer/tumor therapy is everywhere. Only recently have graphene, MXenes, and BPNSs been involved in studies exploring their role in AKI.

#### Black phosphorus nanosheets

Black Phosphorus Nanosheets (BPNSs) show great potential in many fields, such as photothermal/photodynamic therapy [167, 168]. However, as one of the most biologically active nanomaterials, BPNSs have a strong chemical reaction capacity to ROS. The patchy DNA framework enables renal targeted drug delivery. Since BPNSs have the same geometry, this can guide the passive transport of BPNSs to the kidney. BPNSs are easily oxidized to phosphorus oxides in the presence of water, light, and oxygen, which may help to lower cellular ROS [169].

**Fig.7:**
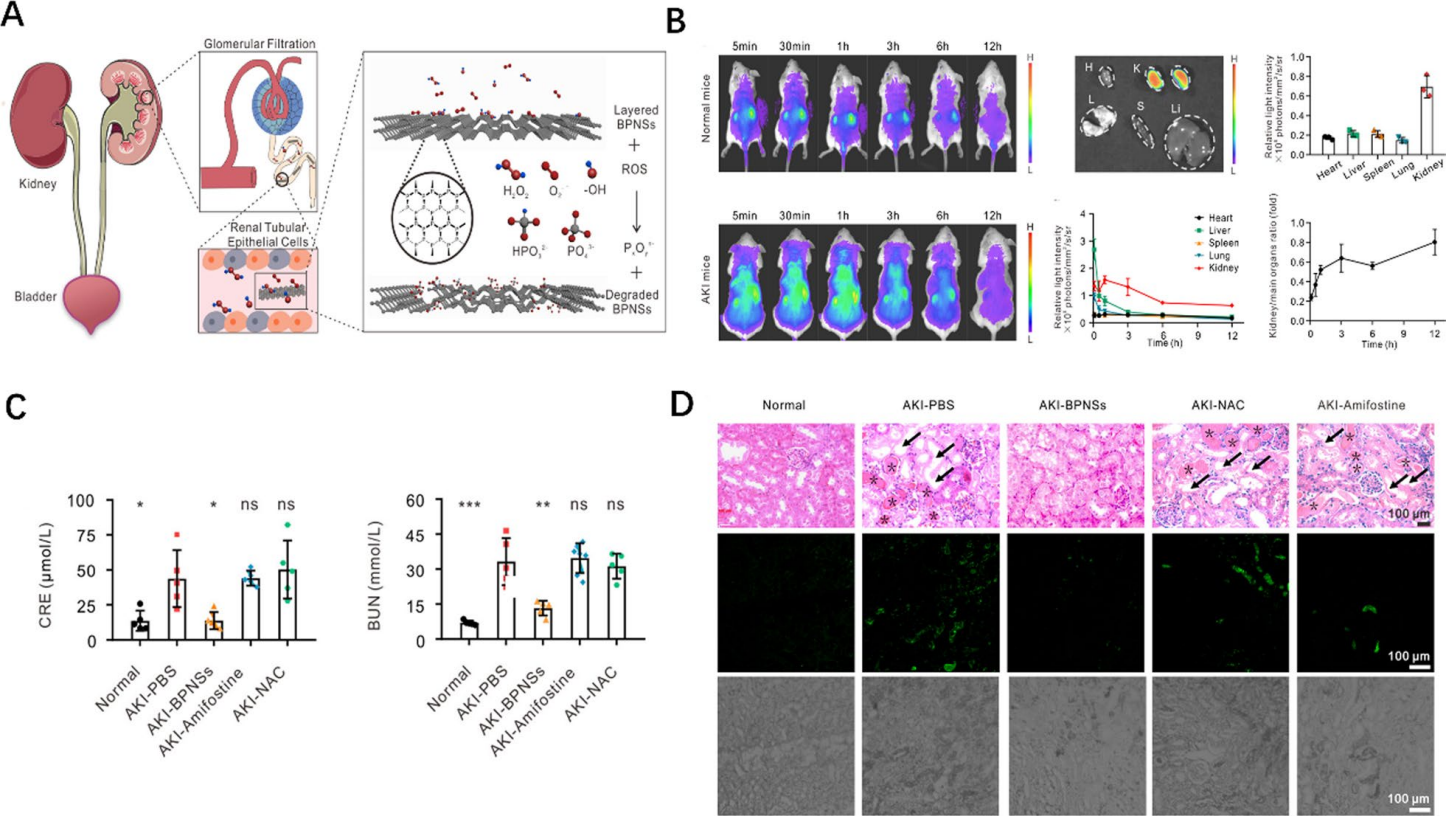
**A** Schematic diagram of the mechanism of BPNSs in the treatment of AKI. **B** Biodistribution of BPNSs in healthy mice and AKI mice. **C** Blood biochemical index from each group after treatment for AKI. **D** H&E staining of kidney tissues and fluorescence images obtained by in situ TUNEL assay from each treatment group. Reprinted with permission from [149]

Hou et al. [149] established a new delivery platform based on BPNSs for removing excessive ROS in injured kidney (Fig. 7A). Cell experiments proved the ROS clearance of BPNSs in HEK 293 cells. BPNSs also exhibited passive targeted aggregation to the kidney in both normal and AKI mice. In AKI mice, 1 h after injection, BPNSs began to transport to the kidney rapidly, and the maximum renal uptake signal appeared at 1 h, and the signal weakened 12 h after injection. Cy5 signal accumulated slowly in AKI mice, indicating that BPNSs inhibited renal clearance rate and increased systemic retention (Fig. 7B). Further studies showed that BPNSs could effectively improve the biochemical blood indexes of AKI mice and reduce the apoptosis of tissue cells. Histopathological changes confirmed the therapeutic effect of BPNSs (Fig. 7C and D). Furthermore, unlike traditional nanomedicines, BPNSs does not have a payload, making their application much more accessible. Most importantly, without cytotoxicity, BPNSs can be degraded to phosphorus oxide after treatment. This study suggests that BPNSs can act as a shape-dependent transporter targeted by the kidney and as a protector against excessive ROS damage.

#### MXenes

**Fig. 8:**
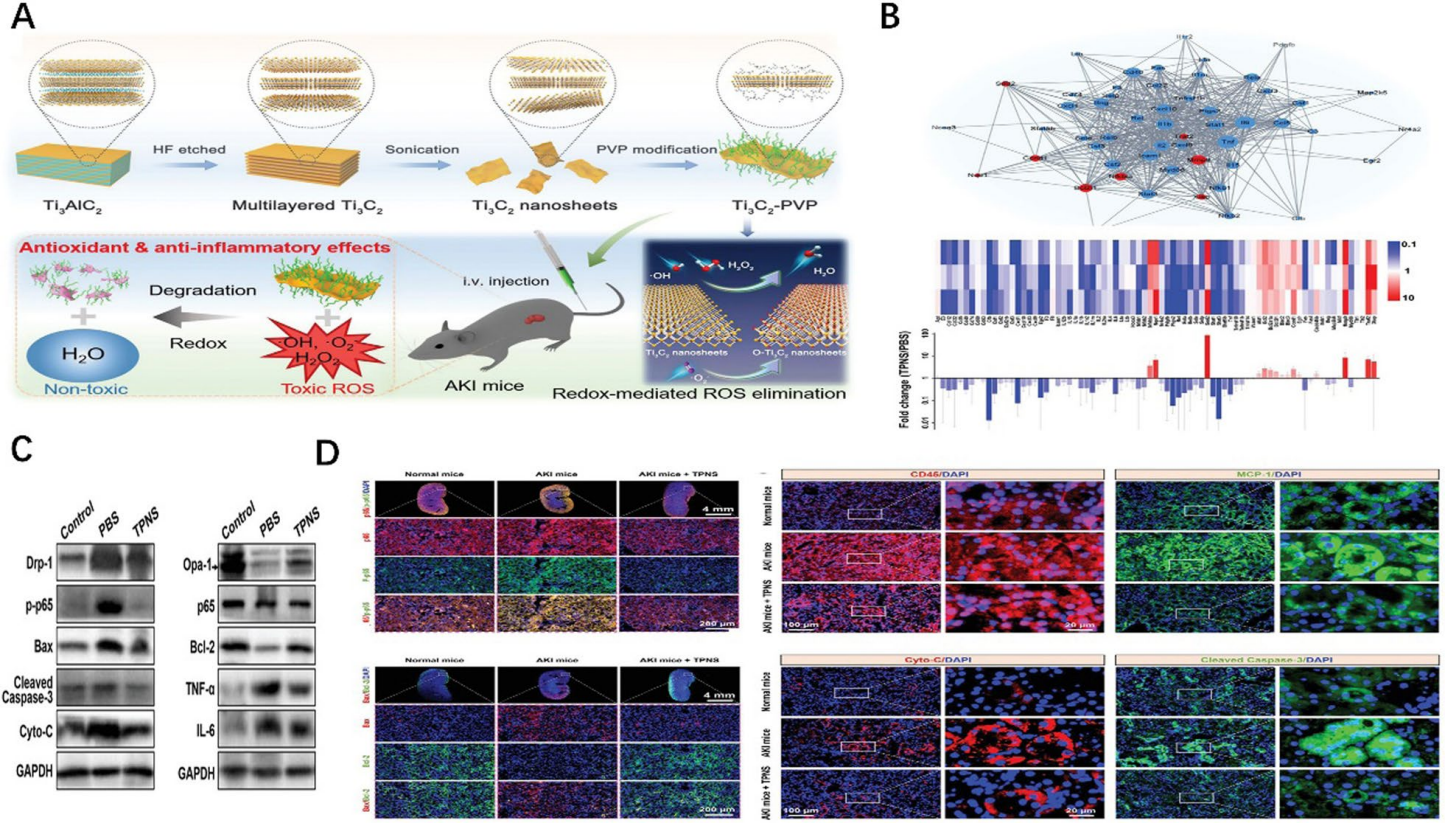
**A** Schematic diagram of TPNS synthesis and antioxidant nanoplatform for AKI therapy. **B** PCR array heat map and fold change of differentially expressed genes; PPI network of differentially‐expressed genes related to the NF-κB signaling pathway. **C** Western blotting analysis of protein levels in renal tissues from each treatment group. **D** Immunofluorescence sections of renal tissues of different treatment groups Reprinted with permission from [150]

As 2D material, MXenes show great potential in the biomedical field and can be used as a promising therapeutic nanomedicine [170–172]. Zhao et al. [150] reported a novel non-enzymatic antioxidant approach based on ultra-thin Ti_3_C_2_-PVP nanosheets (TPNSs) for AKI treatment (Fig. 8A-–D). Ti_3_C_2_ nanosheets were modified by polyvinylpyrrolidone (PVP) to form Ti_3_C_2_-PVP nanosheets, which improved the colloidal stability of Ti_3_C_2_ nanosheets under physiological conditions. 2D Ti_3_C_2_ MXenes have strong chemical reactivity to ROS. In addition, Ti_3_C_2_ MXenes have enzyme/H_2_O_2_ responsive biodegradability, which has a low risk of adverse reactions after treatment [173, 174]. TPNSs also had excellent biodegradability. In vitro experiments confirmed that TPNSs had good scavenging ability on H_2_O_2_, ·O_2_^−^, ·OH, and ABTS radical, and confirmed that TPNSs had a good antioxidant performance. Fluorescence imaging results revealed that TPNSs were preferentially accumulated in the kidney. In vivo experiments, TPNSs showed a superior therapeutic effect by reducing sCr, BUN, and histopathological structure, significantly reducing ROS levels and inhibiting the expression of pro-inflammatory cytokines. Excessive ROS can cause oxidative stress-induced tissue damage, leading to severe inflammatory responses through the NF-κB signaling pathway [175]. Transcriptome analysis showed that TNF-α, IL-2, IL-6, IL-1β, and other important genes related to inflammatory factors were significantly down-regulated after TPNSs treatment, suggesting that TPNSs may be involved in antioxidant and anti-inflammatory protection by inhibiting NF-κB signaling pathway. Therefore, Ti_3_C_2_ MXene is an antioxidant with broad-spectrum ROS scavenging ability and can be used for the treatment of AKI.

#### Graphene and its derivatives

Graphene and its derivatives have been proposed for biomedical applications due to their small size and large specific surface area. Graphene oxide (GO) is a graphene derivative, a new 2D honeycomb carbon-based material. It is a carrier for therapeutic proteins due to its biocompatibility and low toxicity. Because of the large number of oxygen-containing hydrophilic functional groups, including phenol and carboxyl groups, at the edges of GO, the ability to suspend stably in water with oxygen-hydrophilic groups also contributes to the unique physical and chemical properties of GO. A large number of hydroxyl groups on GO’s surface increases its biocompatibility, large surface volume ratio, and particular surface morphology, which enable it to adsorb small molecules and extracellular matrix (ECM) proteins [176, 177].

Foroutan et al. [151] used GO to enhance the efficacy of bone marrow mesenchymal stem cells for AKI treatment. The authors found that the GO surface improved the uptake of bone marrow mesenchymal stem cells. It enabled bone marrow mesenchymal stem cells to access and interact with damaged and healthy kidney stem cells. In addition, GO enhanced interactions between cells and the extracellular matrix.

In another study, Fu et al. [152] reported the therapeutic effect of Fasudil (FSD) hybrid suspended graphene oxide-bovine serum albumin (GO-BSA) bio-composite on severe renal injury in a septicemia model. After intraperitoneal injection of GO-BSA in rats, biomedical examination showed that BUN and sCr were significantly reduced, indicating that GO-BSA could repair acute kidney injury. GO-BSA expands the repair rate by adsorbing ECM proteins and promoting their exchange into severely damaged renal tissue. GO-BSA material may improve the rate of achievement of FSD, conveys in intense renal damage caused by septicopyemia.

## Conclusion and prospects

AKI treatment remains a severe problem due to its complex etiology. Nanomaterials used in the treatment of acute renal damage have recently attracted increasing attention. This review article discusses the pathogenesis of AKI, the current advancement of various novel nanomaterials based on 0D, 1D, and 2D in the treatment of AKI, and the benefits of various nanomaterials.

LDNs hold unique advantages in the treatment of AKI. 0D nanomaterials have a tiny particle size, rapid renal clearance, high biocompatibility, and extensive ROS scavenging capability. Because of its large surface-to-volume ratio, it can be loaded with drugs and tethered to proteins, antibodies, or other biological species. 1D materials can protect and selectively deliver drugs such as RNA and protein to the kidney. They excellent mechanical properties and good biocompatibility, and show good glomerular filtration and elimination curves. Due to the huge surface area and excellent physicochemical properties, 2D materials can absorb many drug molecules for AKI treatment and are able to preferentially accumulate in the kidney. Some 2D nanomaterials, such as BPNSs and Mxenes, can also function as ROS scavengers themselves to mitigate AKI.

However, some challenges remain before LDNs can be used in clinical settings. It is critical to note that animal models differ from human kidneys, and the long-term presence of nanomaterials in humans may have unidentified adverse effects. Detailed toxicity and long-term biosafety studies are required before they can be implemented in clinical settings. LDNs used in human applications must have excellent biocompatibility, degradation, and low toxicity. Meanwhile, to achieve low-cost production and high storage stability, the preparation of LDNs requires simple and feasible preparation steps and production technology.

In the future, with the further understanding and development of renal structure, pathophysiological mechanism and molecular pharmacology, as well as the in-depth study of innovative drug carriers, drug delivery system based on LDNs will shine in the treatment of AKI.

## Data Availability

Not applicable.
